# Supplementary material for: Priority areas to protect mangroves and maximise ecosystem services
Source: Nat Commun. 2023 Sep 21;14:5863. doi: 10.1038/s41467-023-41333-3 (PMC10514197; doi:10.1038/s41467-023-41333-3)
Supplement: Supplementary file 1 — Supplementary Information [file 41467_2023_41333_MOESM1_ESM.pdf]

Supplementary Information

**Mangrove plant species**	*Acanthus ebracteatus, Acanthus volubilis, Acrostichum aureum, Acrostichum danaeifolium, Acrostichum speciosum, Aegialitis annulata, Aegialitis rotundifolia, Aegiceras corniculatum, Aegiceras floridum, Aglaia cucullata, Avicennia alba, Avicennia bicolor, Avicennia germinans, Avicennia integra, Avicennia marina, Avicennia officinalis, Avicennia schaueriana, Brownlowia argentata, Brownlowia tersa, Bruguiera cylindrica, Bruguiera exaristata, Bruguiera gymnorrhiza, Bruguiera hainesii, Bruguiera parviflora, Bruguiera sexangula, Camptostemon philippinense, Camptostemon schultzii, Ceriops australis, Ceriops decandra, Ceriops tagal, Ceriops zippeliana, Conocarpus erectus, Dolichandrone spathacea, Excoecaria agallocha, Excoecaria indica, Heritiera fomes, Heritiera globosa, Heritiera littoralis, Kandelia candel, Kandelia obovata, Laguncularia racemosa, Lumnitzera littorea, Lumnitzera racemosa, Mora oleifera, Nypa fruticans, Osbornia octodonta, Pelliciera rhizophorae, Pemphis acidula, Phoenix paludosa, Rhizophora apiculata, Rhizophora mangle, Rhizophora mucronata, Rhizophora racemosa, Rhizophora samoensis, Rhizophora stylosa, Scyphiphora hydrophylacea, Sonneratia alba, Sonneratia apetala, Sonneratia caseolaris, Sonneratia griffithii, Sonneratia lanceolata, Sonneratia ovata, Tabebuia palustris, Xylocarpus granatum, Xylocarpus moluccensis*
**Marine provinces**	Agulhas, Andaman, Bay of Bengal, Central Polynesia, East Central Australian Shelf, Eastern Coral Triangle, Galapagos, Gulf of Guinea, Hawaii, Java Transitional, North Brazil Shelf, Northeast Australian Shelf, Northern New Zealand, Northwest Australian Shelf, Red Sea and Gulf of Aden, Sahul Shelf, Somali/Arabian, South China Sea, South Kuroshio, Southeast Australian Shelf, Southern New Zealand, Southwest Australian Shelf, Sunda Shelf, Tropical East Pacific, Tropical Northwestern Atlantic, Tropical Northwestern Pacific, Tropical Southwestern Atlantic, Tropical Southwestern Pacific, Warm Temperate Northeast Pacific, Warm Temperate Northwest Atlantic, Warm Temperate Northwest Pacific, Warm Temperate Southwestern Atlantic, West African Transition, West and South Indian Shelf, West Central Australian Shelf, Western Coral Triangle, Western Indian Ocean
**Biophysical typology**	Delta, Estuary, Lagoon, Open Coast, Terrigenous, Carbonate

Supplementary Table 1 – List of the biodiversity data used.

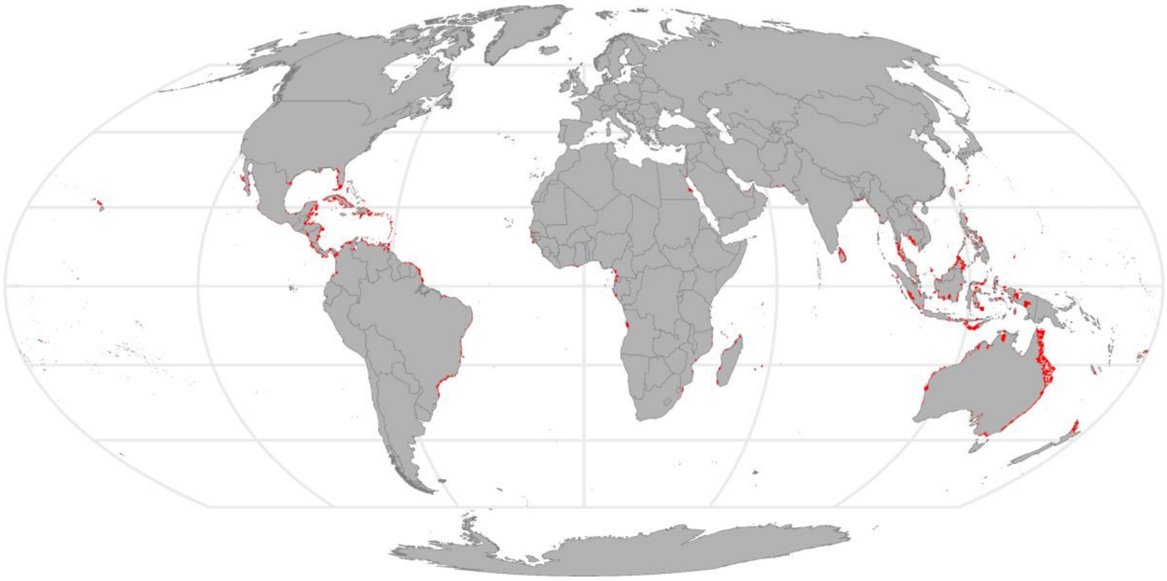

Supplementary Fig. 1 – Protected areas (IUCN categories I-IV) that intersect global mangrove distribution (red colour).

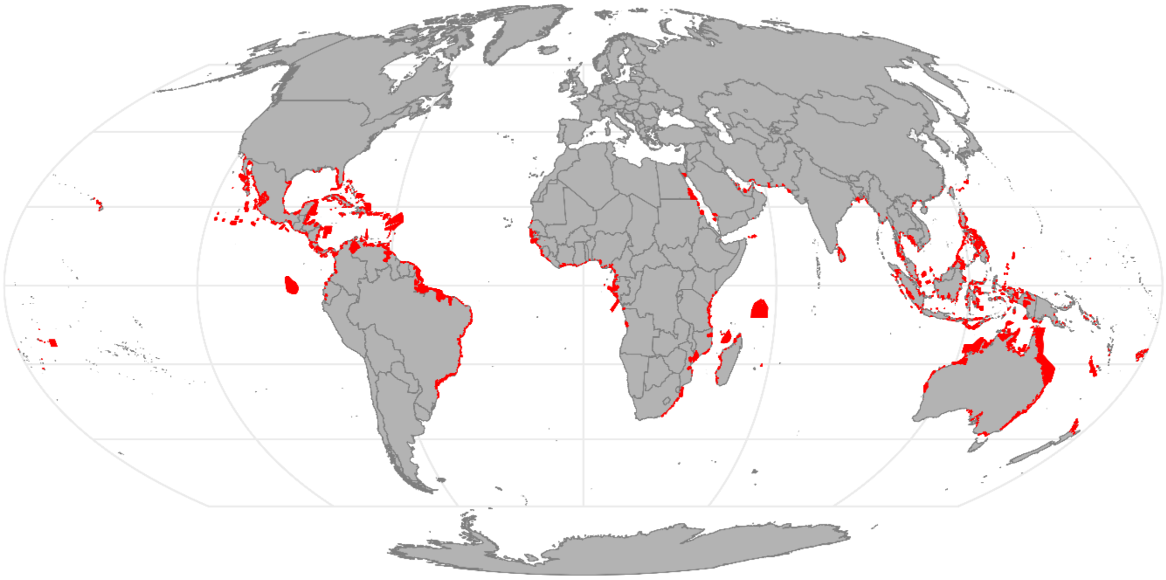

Supplementary Fig. 2 – All protected areas (IUCN categories I-VI and unclassified) that intersect global mangrove distribution (red colour).

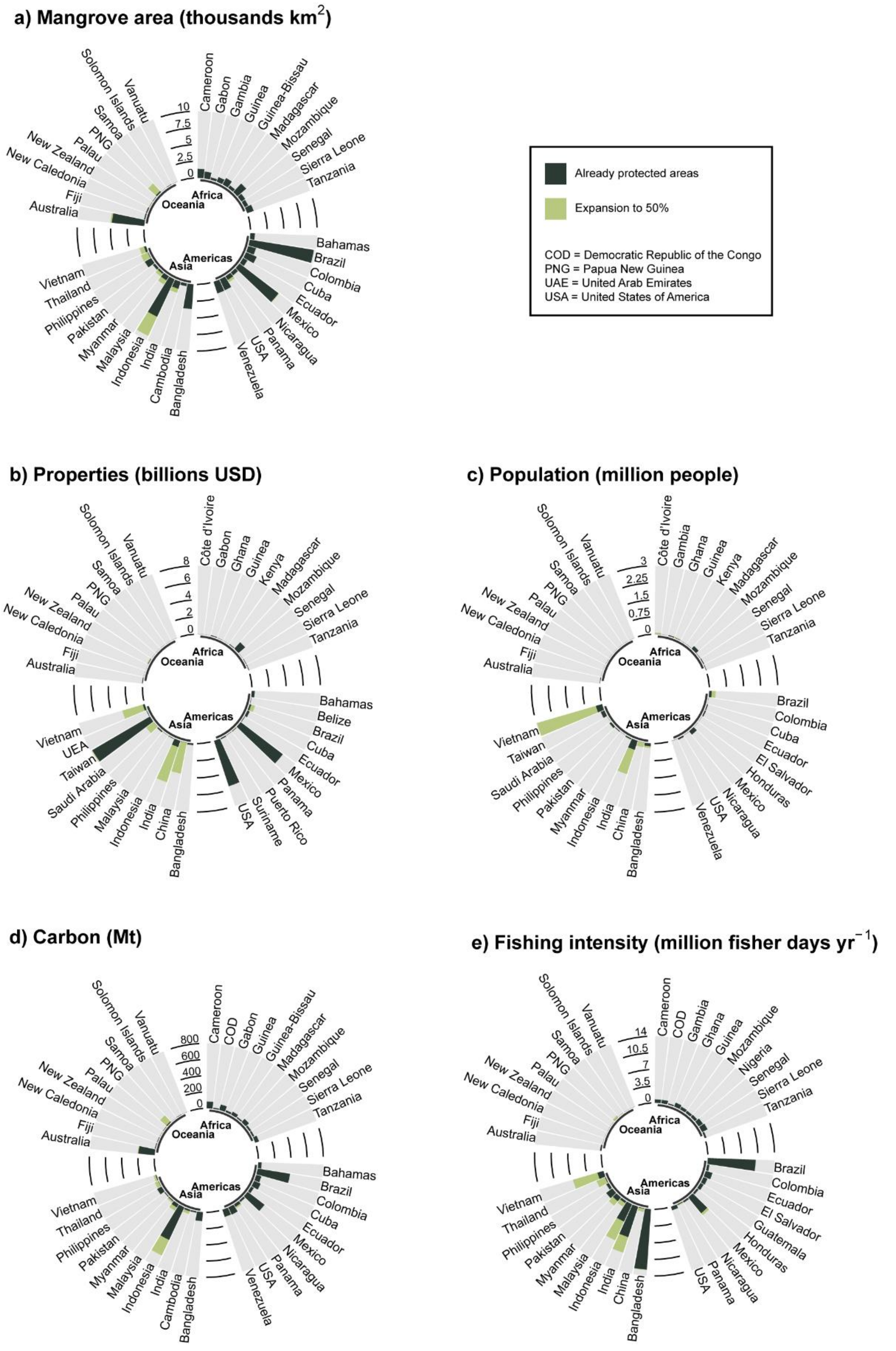

Supplementary Fig. 3 – Cumulative mangrove area and cumulative ecosystem services, by continent and country, for current protected areas (IUCN I-VI and unclassified) and for priority areas added to the current system. a) Mangrove area and **b-e)** ecosystem services provided by current protected areas (dark shade) and priority areas to expand the current system to 50% coverage (light shade) resulting from a prioritisation that maximises protection of

biodiversity and ecosystem services. Only the top-10 countries per continent are shown: **a)** by mangrove area and **b-e)** by amount of ecosystem services provided.

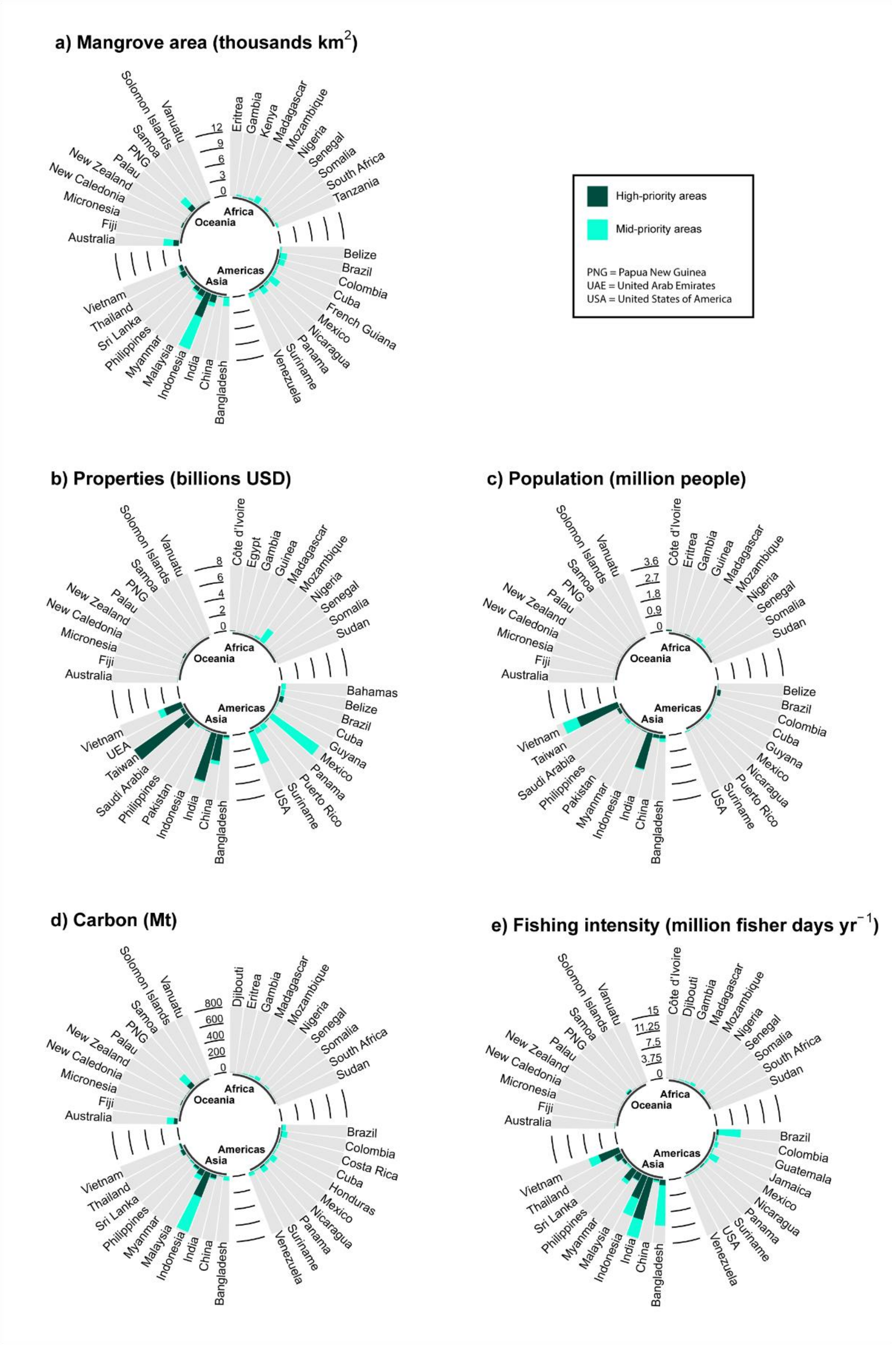

Supplementary Fig. 4 – Cumulative mangrove area and cumulative ecosystem services, by continent and country, for an optimised prioritisation for protection of biodiversity and ecosystem services. a) Mangrove area and **b-e)**

ecosystem services provided by the high-priority areas for protection (dark shade) and the mid-priority areas (light shade) resulting from a prioritisation that maximises protection of biodiversity and ecosystem services. Only the top-10 countries per continent are shown: **a)** by mangrove area and **b-e)** by amount of ecosystem services provided.

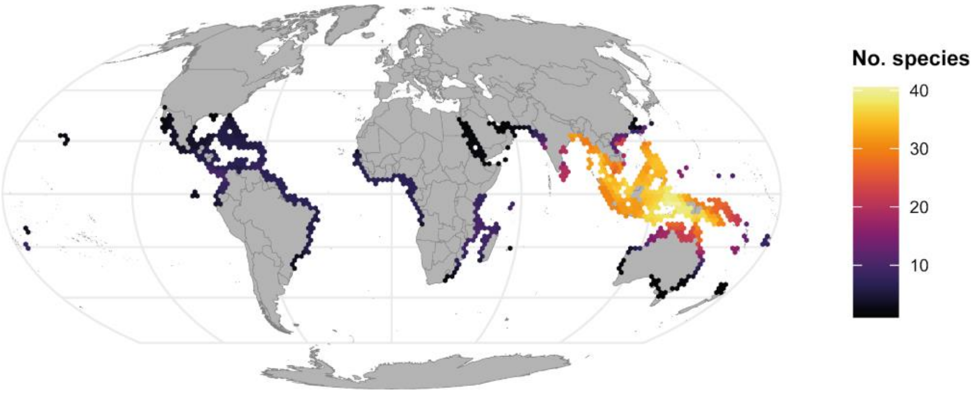

Supplementary Fig. 5 – Distribution of species richness. Global distribution of total mangrove species considered in the analysis. Data are aggregated at 200-km alongshore resolution for visualisation.

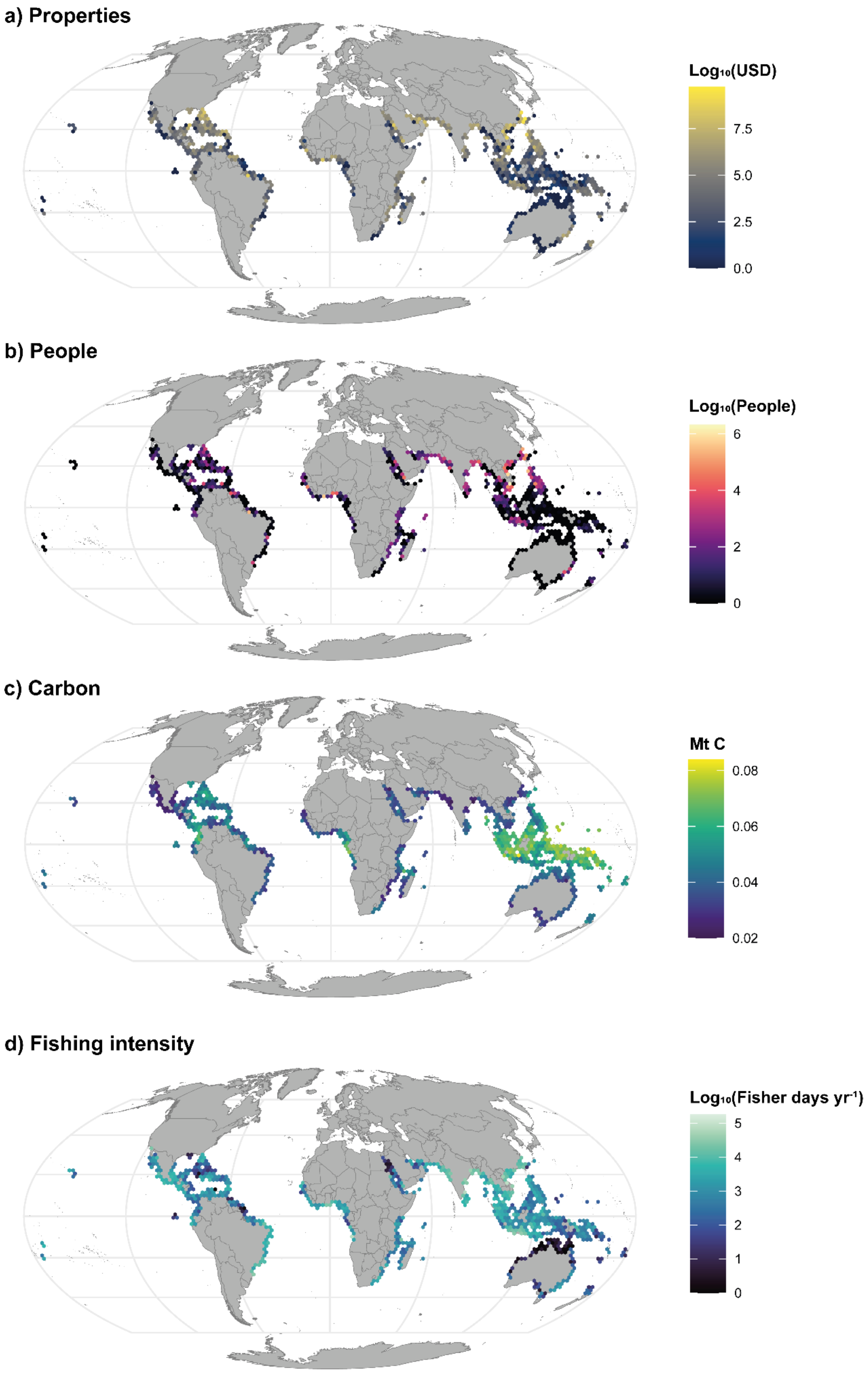

Supplementary Fig. 6 – Distribution of ecosystem services. Global distribution of: a) value of properties and b) number of people protected by mangroves from Menéndez et al.[1]; c) carbon stored by mangroves from Simard et al.[2]

and Sanderman et al.[3]; and d) fishing intensity in mangroves from zu Ermgassen et al.[4]. Data are aggregated at a 200-km alongshore resolution for visualisation.

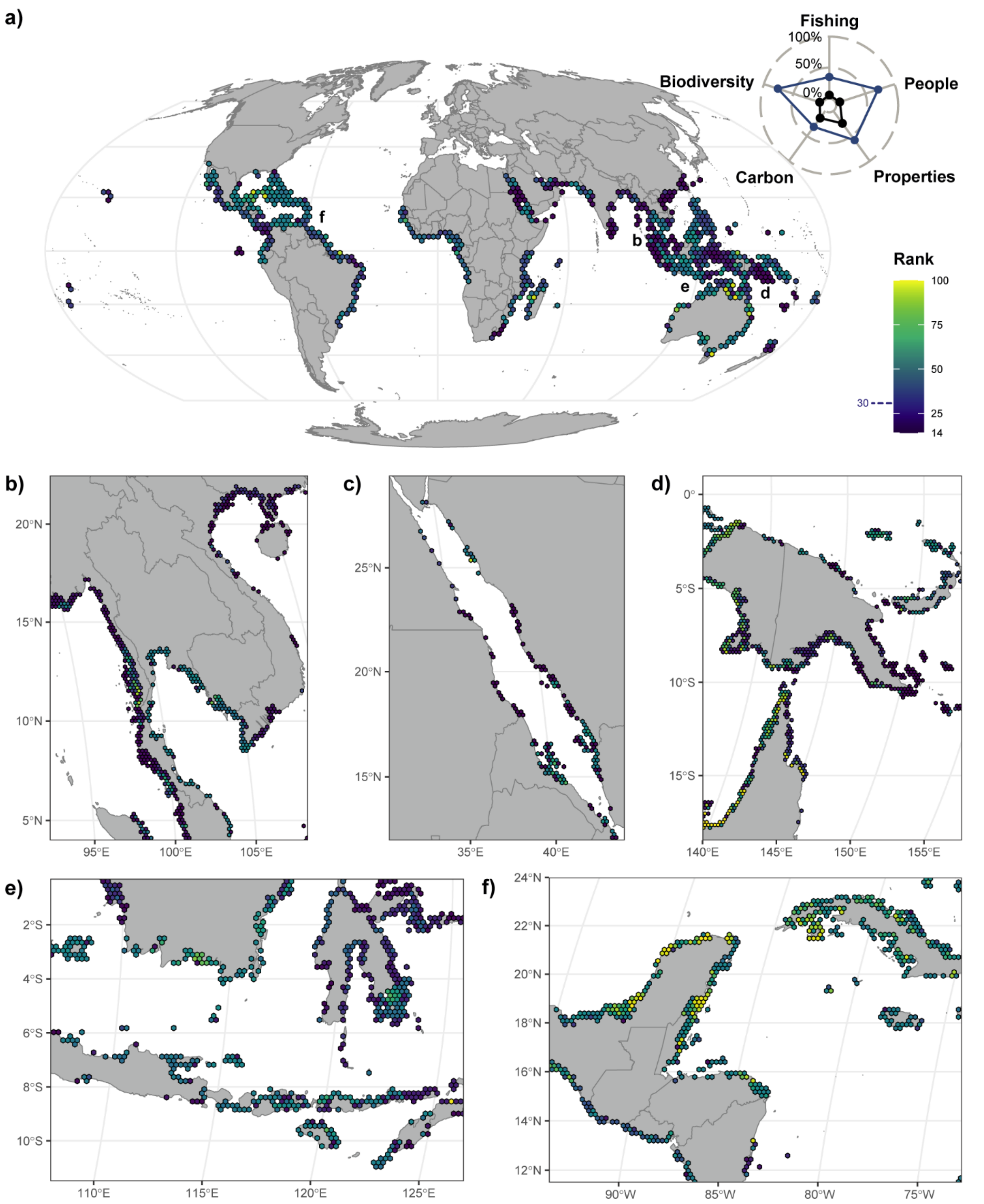

Supplementary Fig. 7 – Global mangrove priority areas for protection expanding the current strictly-protected area system. a) Priority map, with hexagonal bins aggregated at a resolution of 200-km alongshore for visualisation, shows the median value of the rank of the planning units in the selection (the lower the rank, the more important the

planning unit is in terms of protecting biodiversity and delivering ecosystem services). Planning units currently strictly-protected are not included in the maps. The radar plot shows the percentage of ecosystem services protected and the percentage of conservation targets reached in the current strictly-protected areas (in black), and in priority areas to expand protection to 30% of the global mangrove area (same colour scale of the map). Insets maps (planning units at the analysis resolution of 20-km alongshore) show results for: **b)** Southeast Asia; **c)** The Red Sea; **d)** West Papua, Papua New Guinea, Northern Australia; **e)** Indonesia; and **f)** Mesoamerica and Cuba.

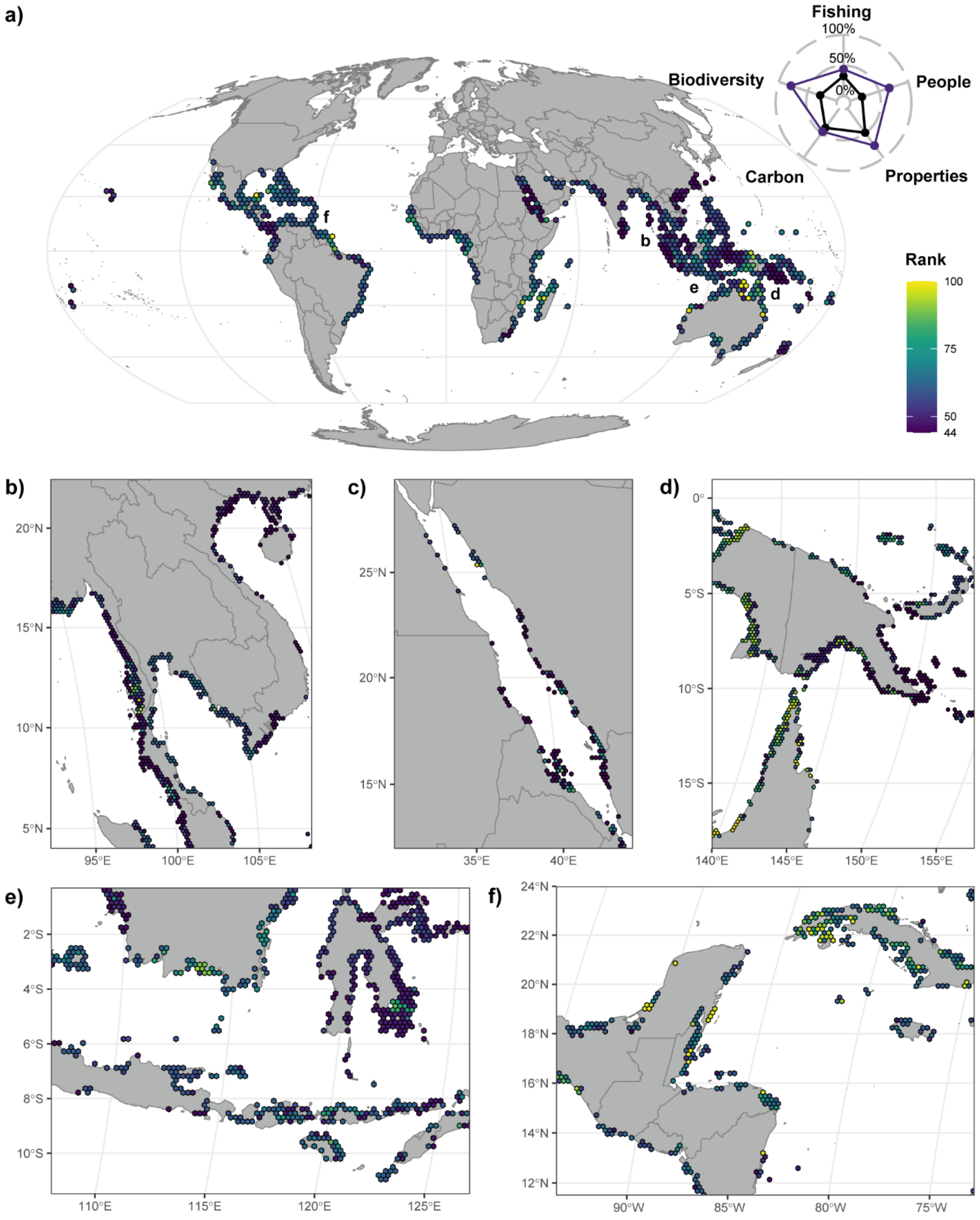

Supplementary Fig. 8 – Global mangrove priority areas for protection expanding the current protected area system (IUCN I-VI and unclassified). a) Priority map, with hexagonal bins aggregated at a resolution of 200-km alongshore for visualisation, shows the median value of the rank of the planning units in the selection (the lower the rank, the more important the planning unit is for protecting biodiversity and delivering ecosystem services). The radar plot shows the

percentage of ecosystem services protected and the percentage of conservation targets reached in the current protected areas (in black), and in priority areas to expand protection to 50% of the global mangrove area (same colour scale as the map). Inset maps (planning units at the analysis resolution of 20-km alongshore) show results for: **b)** Southeast Asia; **c)** The Red Sea; **d)** West Papua, Papua New Guinea, Northern Australia; **e)** Indonesia; and **f)** Mesoamerica and Cuba.

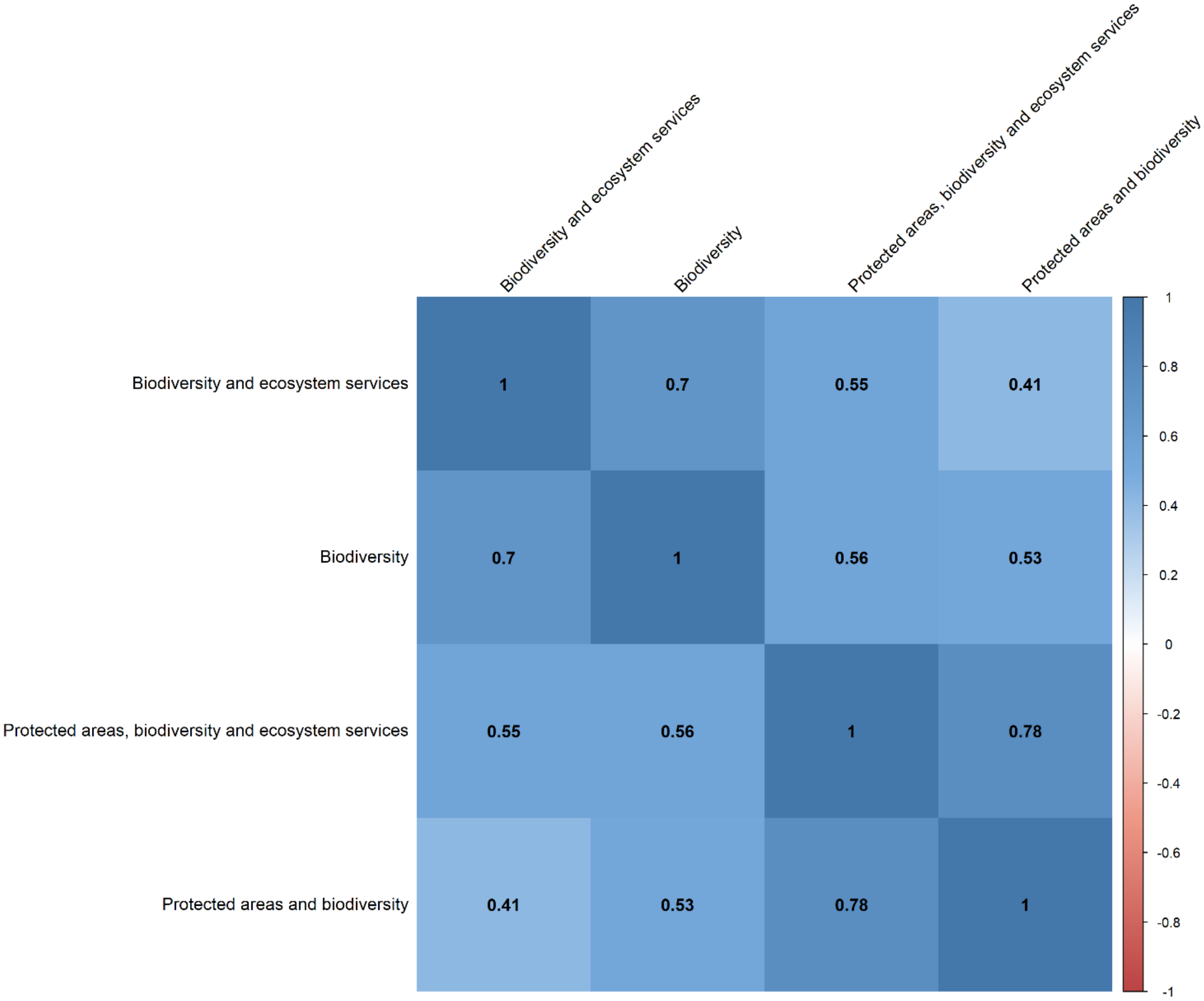

Supplementary Fig. 9 – Cohen's Kappa correlation plot. Degree of agreement between the results of the prioritisations to protect 30% of the global mangrove area.

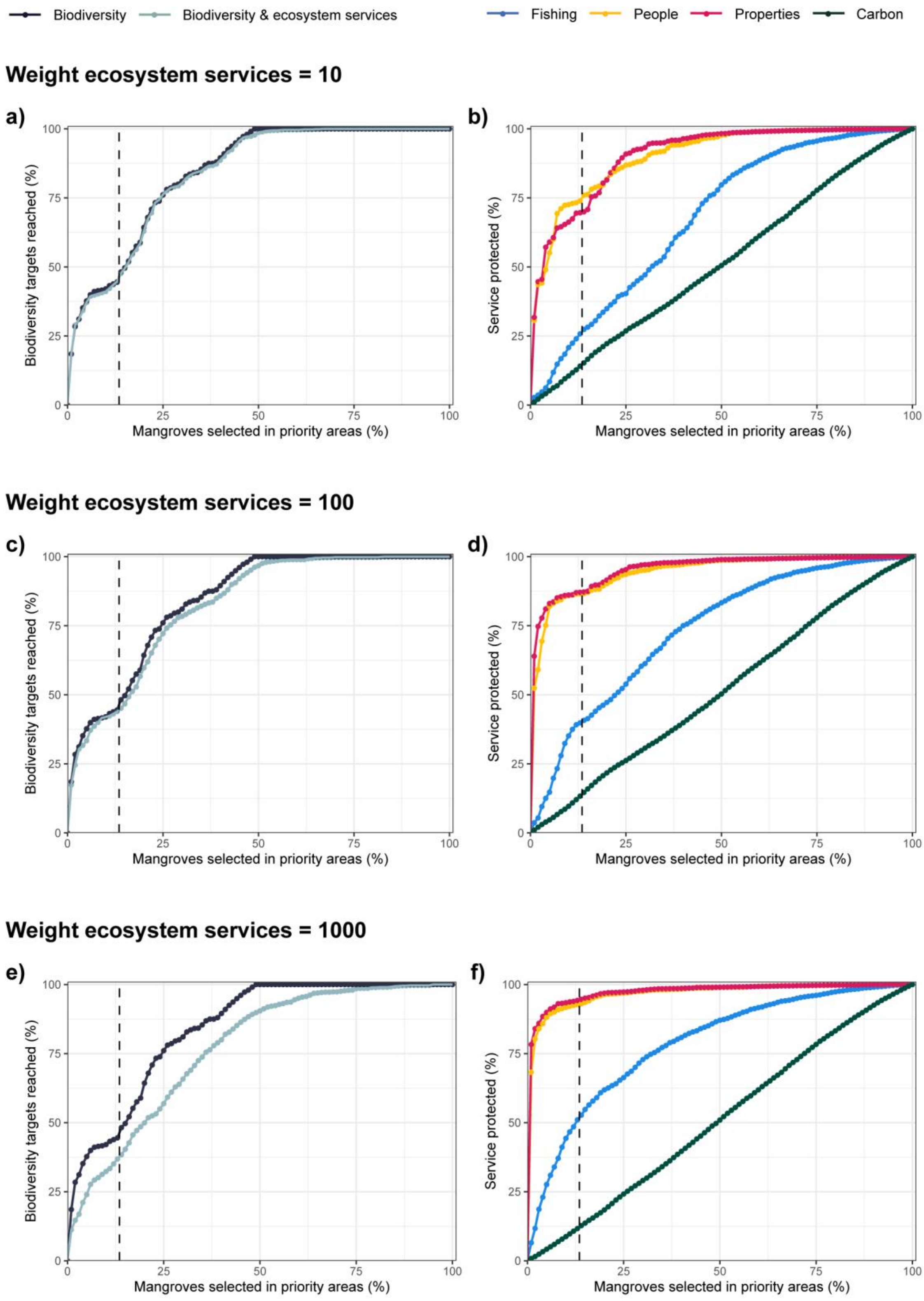

Supplementary Fig. 10 – Variation in species conservation targets reached and ecosystem services conserved when changing ecosystem services weights, in a series of prioritisation based on incremental area budgets (*i.e.*, increasing the area of mangroves protected). **a, c, e)** Conservation targets reached (%) in a spatial prioritisation optimised for biodiversity alone, or on biodiversity and ecosystem services, and **b, d, f)** increase in the percentage of the total of

each ecosystem service protected for a spatial prioritisation optimised focusing on biodiversity and ecosystem services. Prioritisations were run varying the ecosystem services weights: **a, b)** 10; **c, d)** 100 and **e, f)** 1,000. The percentage of mangrove area currently protected is indicated by a dashed vertical line.

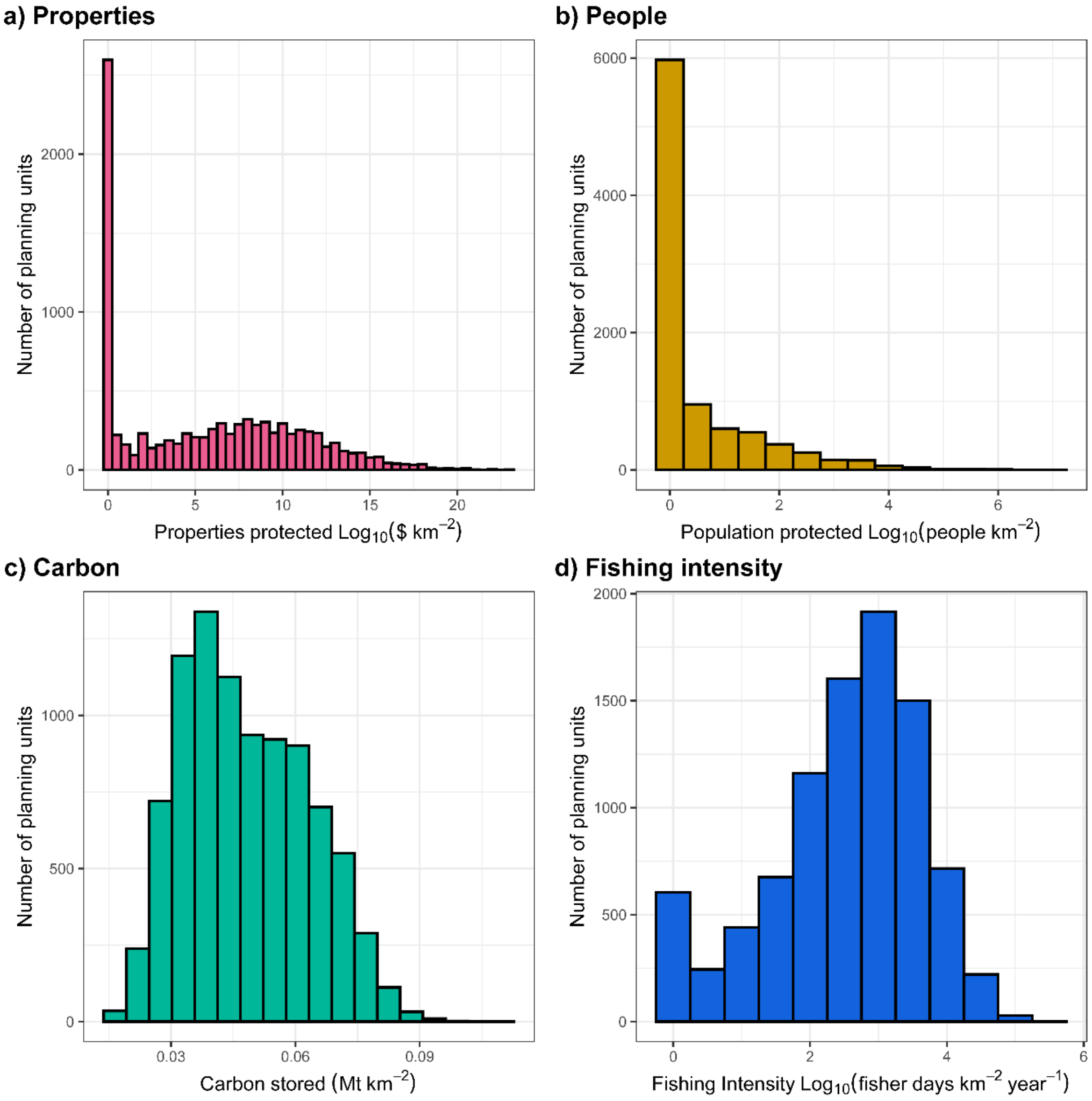

Supplementary Fig. 11 – Histograms of the ecosystem services provided by mangroves in each planning unit. Histograms for the mean value of **a)** properties protected, **b)** population protected, **c)** carbon stored and **d)** fishing intensity.

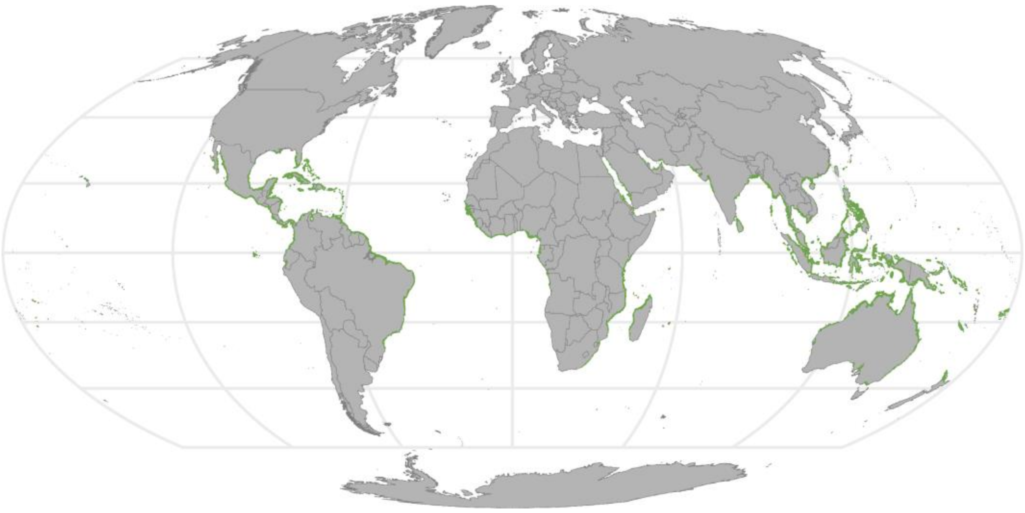

Supplementary Fig. 12 – Distribution of mangroves aggregated at 20-km alongshore resolution (green colour).

High-resolution figures of the global maps are available at[5]:

https://doi.org/10.5281/ZENODO.8272951

Supplementary References

1. Menéndez, P., Losada, I. J., Torres-Ortega, S., Narayan, S. & Beck, M. W. The Global Flood Protection Benefits of Mangroves. *Sci. Rep.* **10**, 4404 (2020).
2. Simard, M. *et al.* Mangrove canopy height globally related to precipitation, temperature and cyclone frequency. *Nat. Geosci.* **12**, 40–45 (2019).
3. Sanderman, J. *et al.* A global map of mangrove forest soil carbon at 30 m spatial resolution. *Environ. Res. Lett.* **13**, 055002 (2018).
4. zu Ermgassen, P. S. E. *et al.* Reprint of : Fishers who rely on mangroves: Modelling and mapping the global intensity of mangrove-associated fisheries. *Estuar. Coast. Shelf Sci.* **248**, 107159 (2021).
5. Dabalà, A. *et al.* Priority areas to protect mangroves and maximise ecosystem services - code release v.1.0.1. (2023) doi:10.5281/ZENODO.8272951.
