# Supplementary material for: Priority areas to protect mangroves and maximise ecosystem services
Source: Nat Commun. 2023 Sep 21;14:5863. doi: 10.1038/s41467-023-41333-3 (PMC10514197; doi:10.1038/s41467-023-41333-3)
Supplement: Supplementary file 3 — Reporting Summary [file 41467_2023_41333_MOESM3_ESM.pdf]

nature portfolio

Corresponding author(s): Dabalà Alvise

Last updated by author(s): Aug 14, 2023

Reporting Summary

Nature Portfolio wishes to improve the reproducibility of the work that we publish. This form provides structure for consistency and transparency in reporting. For further information on Nature Portfolio policies, see our Editorial Policies and the Editorial Policy Checklist.

Statistics

For all statistical analyses, confirm that the following items are present in the figure legend, table legend, main text, or Methods section.

n/a	Confirmed	
☐	☒	The exact sample size (*n*) for each experimental group/condition, given as a discrete number and unit of measurement
☒	☐	A statement on whether measurements were taken from distinct samples or whether the same sample was measured repeatedly
☒	☐	The statistical test(s) used AND whether they are one- or two-sided<br>*Only common tests should be described solely by name; describe more complex techniques in the Methods section.*
☒	☐	A description of all covariates tested
☒	☐	A description of any assumptions or corrections, such as tests of normality and adjustment for multiple comparisons
☐	☒	A full description of the statistical parameters including central tendency (e.g. means) or other basic estimates (e.g. regression coefficient) AND variation (e.g. standard deviation) or associated estimates of uncertainty (e.g. confidence intervals)
☒	☐	For null hypothesis testing, the test statistic (e.g. *F*, *t*, *r*) with confidence intervals, effect sizes, degrees of freedom and *P* value noted<br>*Give P values as exact values whenever suitable.*
☒	☐	For Bayesian analysis, information on the choice of priors and Markov chain Monte Carlo settings
☒	☐	For hierarchical and complex designs, identification of the appropriate level for tests and full reporting of outcomes
☒	☐	Estimates of effect sizes (e.g. Cohen's *d*, Pearson's *r*), indicating how they were calculated

Our web collection on statistics for biologists contains articles on many of the points above.

Software and code

Policy information about availability of computer code

Data collection	No software was used for the data collection.
Data analysis	All analysis was conducted in R statistical computing environment (version 4.2.3). All prioritisations were completed using the prioritizr R package and Gurobi (https://www.gurobi.com). Code to run the analysis in this study is currently available at: https://github.com/AlviDab/SpatialPrioritisation_MangrovesEcosystemBenefits. The code is archived in a Zenodo digital repository: https://doi.org/10.5281/ZENODO.8272951.

For manuscripts utilizing custom algorithms or software that are central to the research but not yet described in published literature, software must be made available to editors and reviewers. We strongly encourage code deposition in a community repository (e.g. GitHub). See the Nature Portfolio guidelines for submitting code & software for further information.

Data

Policy information about availability of data

All manuscripts must include a data availability statement. This statement should provide the following information, where applicable:

- Accession codes, unique identifiers, or web links for publicly available datasets
- A description of any restrictions on data availability
- For clinical datasets or third party data, please ensure that the statement adheres to our policy

Datasets generated analysed during the current study are available online. The global map of mangroves is available for download from the Global Mangrove Watch

website (https://www.globalmangrovewatch.org/). IUCN distribution of mangrove species is available at: https://www.iucnredlist.org/resources/spatial-data-download. The global biophysical mangrove typology and the marine provinces of the world are available for download from the Ocean Data Viewer (https://data.unep-wcmc.org/). The ecosystem services layers can be downloaded for aboveground carbon (https://daac.ornl.gov/cgi-bin/dsviewer.pl?ds_id=1665), soil carbon (https://zenodo.org/record/1469348#.Yv9HDcJBy3A), and coastal protection (https://osf.io/ecs4p/). For fishing intensity data, please contact the authors of the original article (https://doi.org/10.1016/j.ecss.2020.106975). Data on protected areas can be downloaded from the WDPA database (https://www.protectedplanet.net/). Global coastline data can be downloaded using the rnaturalearth package. Exclusive Economic Zones boundaries are available at: https://www.marineregions.org/sources.php#marbound. Source data are provided with this paper. Datasets generated during the current study are currently available at: https://doi.org/10.5281/ZENODO.8272951.

Research involving human participants, their data, or biological material

Policy information about studies with human participants or human data. See also policy information about sex, gender (identity/presentation), and sexual orientation and race, ethnicity and racism.

Reporting on sex and gender	This study does not include any human reseach participants.
Reporting on race, ethnicity, or other socially relevant groupings	This study does not include any human reseach participants.
Population characteristics	This study does not include any human reseach participants.
Recruitment	This study does not include any human reseach participants.
Ethics oversight	This study does not include any human reseach participants.

Note that full information on the approval of the study protocol must also be provided in the manuscript.

Field-specific reporting

Please select the one below that is the best fit for your research. If you are not sure, read the appropriate sections before making your selection.

☐ Life sciences ☐ Behavioural & social sciences ☒ Ecological, evolutionary & environmental sciences

For a reference copy of the document with all sections, see nature.com/documents/nr-reporting-summary-flat.pdf

Ecological, evolutionary & environmental sciences study design

All studies must disclose on these points even when the disclosure is negative.

Study description	This study identifies priority areas for protection of mangroves biodiversity and ecosystem services.
Research sample	We obtained a fine-scale global distribution map of mangroves from the Global Mangrove Watch (GMW; https://www.globalmangrovewatch.org/). We obtained geographic range data of the 65 most common mangrove species from the IUCN Red List of Threatened Species (https://www.iucnredlist.org/resources/spatial-data-download). Biophysical and biogeographic data were included in the analysis to define a system that ensures the greatest benefits for biodiversity representation, namely: 1) mangrove biophysical typology; 2) and marine provinces of the world (https://data.unep-wcmc.org/). We also obtained data on ecosystem services in mangrove areas, namely: coastal protection of both people and properties (https://osf.io/ecs4p/), carbon sequestration (https://daac.ornl.gov/cgi-bin/dsviewer.pl?ds_id=1665; https://zenodo.org/record/1469348#.Yv9HDcJBy3A), and benefits for fisheries (https://doi.org/10.1016/j.ecss.2020.106975). Protected area data were obtained from the World Database on Protected Areas (WDPA; https://www.protectedplanet.net/). Global coastline data were obtained using the rnaturalearth package. Exclusive Economic Zones boundaries were downloaded at: https://www.marineregions.org/sources.php#marbound.
Sampling strategy	Not applicable.
Data collection	We used data publicly available. Data were downloaded from online databases.
Timing and spatial scale	Data were not collected during this study. We used publicly available global scale mangrove data from 2016.
Data exclusions	No data were excluded from the analysis.
Reproducibility	The code to reproduce the main results is currently available at: https://github.com/AlviDab/SpatialPrioritisation_MangrovesEcosystemBenefits. The code is archived in a Zenodo digital repository: https://doi.org/10.5281/ZENODO.8272951.
Randomization	Not applicable.
Blinding	Not applicable.

Did the study involve field work? ☐ Yes ☒ No

Reporting for specific materials, systems and methods

We require information from authors about some types of materials, experimental systems and methods used in many studies. Here, indicate whether each material, system or method listed is relevant to your study. If you are not sure if a list item applies to your research, read the appropriate section before selecting a response.

Materials & experimental systems

n/a	Involved in the study
☒	☐ Antibodies
☒	☐ Eukaryotic cell lines
☒	☐ Palaeontology and archaeology
☒	☐ Animals and other organisms
☒	☐ Clinical data
☒	☐ Dual use research of concern
☒	☐ Plants

Methods

n/a	Involved in the study
☒	☐ ChIP-seq
☒	☐ Flow cytometry
☒	☐ MRI-based neuroimaging
